# Polymorphonuclear (PMN) elastase in patients after severe traumatic brain injury

**DOI:** 10.1186/s40001-018-0341-x

**Published:** 2018-09-15

**Authors:** Lukas Kurt Postl, Viktoria Bogner, Martijn van Griensven, Marc Beirer, Karl Georg Kanz, Christoph Egginger, Peter Biberthaler, Chlodwig Kirchhoff

**Affiliations:** 1 Department of Trauma Surgery, Klinikum rechts der Isar, Technical University of Munich, Ismaninger Str. 22, 81675 Munich, Germany; 2 Department of Trauma Surgery, Ludwig Maximilians University Munich, 80336 Munich, Germany; 3 Department of Trauma Surgery, Klinikum Passau, 94032 Passau, Germany

**Keywords:** Polymorphonuclear elastase, PMN, Traumatic brain injury, TBI, Alpha 1-proteinase, ELISA

## Abstract

**Background:**

Data on PNM elastase levels in cerebrospinal fluid following traumatic brain injury (TBI) in humans are not available in the literature. Therefore, the aim of this prospective study was to evaluate the dynamics of PMN elastase in the cerebrospinal fluid (CSF) of patients after TBI.

**Methods:**

Patients suffering from isolated, closed TBI, presenting with an initial Glasgow coma score ≤ 8 and with intracerebral hemorrhage on the initial cranial computed tomography scan (performed within 90 min after TBI) were enrolled. CSF and blood samples were obtained immediately, 12 h, 24 h, 48 h, and 72 h after admission. ELISA testing was used to quantify the PMN elastase levels in CSF. In addition, the ratio of CSF albumin to serum albumin was calculated to evaluate the role of the blood–cerebrospinal fluid barrier (BCSFB). As controls, CSF samples were taken from patients receiving spinal anesthesia for elective orthopedic surgery of the lower extremity.

**Results:**

Twenty-three patients meeting the inclusion criteria and ten control patients were enrolled. The PMN elastase showed a significant elevation at 48 and 72 h after TBI. When comparing the PMN elastase levels of patients with intact BCSFB to patients with defective BCSFB, there was no significant difference for the respective observation points.

**Conclusions:**

This is the first study to demonstrate that the PMN elastase levels in CSF significantly increased in the early posttraumatic phase (48 h and 72 h after TBI) in patients. The function of the BCSFB showed no significant influence on the PMN levels.

## Background

Traumatic brain injury (TBI) often affects young adults and is a major cause of persistent neurocognitive impairment in this age group [1]. In general, the severity of the primary traumatic injury and the extent of the secondary biomolecular injury cascades determine the outcome [2]. Since these secondary injuries might be responsible for the development of neurological deficits, they might serve as a potential target in the development of new therapeutic interventions [3]. In this context, it seems crucial to further explore and understand the intracranial processes following TBI [3].

Granulocytes or polymorphonuclear (PMN) leukocytes have granules in their cytoplasm which contain elastase that can be secreted. This so-called PMN elastase is involved in the extracellular immune defense of microorganisms. Its activity in the extracellular space is regulated by the formation of alpha 1-proteinase inhibitor–elastase complexes. Consequently, the level of inhibitor–elastase complexes correlates with the elastase that is actually secreted [4]. Severe inflammatory conditions can lead to an overload of the human immune system and elastase, especially in combination with oxidants (e.g., H_2_O_2_, O_2_ radicals), which can lead to tissue damage.

Under physiological conditions, the cerebrospinal fluid (CSF) is separated from peripheral and cerebral blood flow by the blood–cerebrospinal fluid barrier (BCSFB). When analyzing the dynamics of the PMN elastase in CSF of TBI patients the question arises whether changes in CSF fractions occur due to a disrupted BCSFB or due to other mechanisms. The leukocyte count in CSF is far lower than in peripheral blood. A damaged BCSFB could, therefore, possibly lead to an increase in the number of granulocytes in the CSF via cell leakage due to disturbed blood vessels. Consequently, PMN elastase secretion would be enhanced resulting in an increased level. Thus, it is necessary to take the function of the BCSFB into account when evaluating the PMN elastase.

It is well known that the proteinase PMN elastase plays an important role in acute injury and inflammation [5–8]. However, data on PNM elastase levels in cerebrospinal fluid following TBI in humans are not available in the literature.

Therefore, the aim of the present study was to evaluate the dynamics of PMN elastase in the CSF of patients with TBI from the time of admission until 72 h afterwards. The first null hypothesis was that there was no difference between the CSF levels of patients at the various sample points. Second null hypothesis postulated that there was no difference between the CSF values of the patients (with TBI) and the control group. The influence of the BCSFB was investigated by distinguishing two groups of patients with TBI: those with an intact and those with a defective BCSFB. The level of significance was set to 0.05.

## Patients and methods

### Study design and patient population

Patients suffering from closed, isolated TBI, who presented with an initial Glasgow coma score (GCS) ≤ 8 points (i.e., severe brain injury) and radiological signs of an intracerebral hemorrhage (ICH) on the initial cranial CT scan (performed within 90 min after TBI) were prospectively enrolled. Patients with an untreatable brain swelling and/or brain stem herniation were excluded. A history of pre-existing neurological, malignant or chronic inflammatory disease led to exclusion as well. Written informed consent was obtained as soon as the patient regained consciousness. In case of remaining unconscious, either a legal representative or a next of kin was asked for the (supposed) will of the patient. As controls, CSF samples were taken from patients receiving spinal anesthesia for elective orthopedic surgery of the lower extremity. The study was approved by the University Institutional Review Board (Reference no. 330/03).

### Treatment

Patients were treated in accordance with the guidelines of the Brain Trauma Foundation [9]. An extraventricular drain (TraumaCath, Integra Neurosciences; Plainsboro, USA) was positioned in the frontal horn of the lateral ventricle to obtain CSF for further analysis and to continuously monitor the patient’s intracranial pressure (ICP) [10]. After the procedure, a CT scan was performed to verify the position of the drain. As soon as the ICP remained at a level of maximal 15 mmHg for at least 72 h—without CSF drainage or mannitol administration—the drain was removed.

### Sampling

The first sample was drawn immediately after the placement of the intraventricular drain within 90 ± 45 min after admission to the hospital. Further sampling points were at 12, 24, 48 and 72 h after TBI. Approximately 4 ml of CSF and 5 ml of peripheral serum were collected at each sampling point. 500 µl of liquor and peripheral serum was sent to the department of hematology to determine the respective albumin levels there. For the enzyme-linked immunosorbent assay (ELISA) analysis, the CSF samples were centrifuged for 10 min with 1200*g* at 4 °C. Afterwards, the supernatant was frozen at − 80 °C.

### Analysis of PMN elastase concentration in CSF

An enzyme immunoassay (Milena Biotech, Bad Nauheim, Germany) was used for the quantitative measurement of the human PMN elastase. The assay contained the following components:

1. divisible microplate removable wells, coated with polyclonal antibodies (egg yolk) against PMN elastase;
2. PMN elastase master calibrator in serum/buffer matrix, containing PMN elastase–α1PI complex;
3. PMN elastase controls in serum/buffer matrix;
4. PMN elastase calibrator/sample diluent;
5. enzyme-labeled anti-α1PI antibody, containing polyclonal rabbit antibodies, labeled with horseradish peroxidase;
6. TMB-substrate solution (3,3′,5,5′-tetra-methyl-benzidine) in buffered peroxide solution;
7. stop solution, containing 2 M hydrochloric acid;
8. wash buffer concentrate (10×).

The sampling material was thawed at 20 °C for analysis. Thirty minutes before use, the PMN elastase master calibrator (Milena Biotech, Bad Nauheim, Germany) and the PMN elastase controls (Milena Biotech, Bad Nauheim, Germany) were each reconstituted with 2 ml calibrator/sample diluent (Milena Biotech, Bad Nauheim, Germany). The calibrators were prepared with a twofold dilution series (1000 ng/ml, 500 ng/ml, 250 ng/ml, 125 ng/ml, 62.5 ng/ml, 31.3 ng/ml and 15.6 ng/ml). All patient samples were diluted with a ratio of 1:100 using calibrator/sample buffer (Milena Biotech, Bad Nauheim, Germany). Subsequently, 100 µl of prediluted patient samples, calibrators, and controls was pipetted into the microplate’s wells, according to the assay’s template (Milena Biotech, Bad Nauheim, Germany). This was followed by an incubation period of 60 min at 20 °C on a plate mixer with 400 rpm. Afterwards, the samples were washed four times using 300 µl buffered wash solution (Milena Biotech, Bad Nauheim, Germany). Subsequently, 150 µl of enzyme-labeled anti-α1PI antibodies (Milena Biotech, Bad Nauheim, Germany) was added. The second incubation period of 60 min at 20 °C on a plate mixer with 400 rpm led to the formation of so-called “sandwich” complexes. After repeated fourfold washing using 300 µl buffered wash solution (Milena Biotech, Bad Nauheim, Germany), 200 µl of TMB in buffered peroxide solution (Milena Biotech, Bad Nauheim, Germany) was added. This was followed by an incubation period of 20 min in the dark at 20 °C. 50 µl of 2 M hydrochloric acid was added before the sample was thoroughly mixed. Finally, the produced yellow signal was measured at a wavelength of 450 nm. Using a standard curve, the concentration of the PMN elastase could be determined from the signal.

### Assessment of blood–cerebrospinal fluid barrier (BCSFB) function

Reiber and Felgenhauer were able to show that the ratio of albumin in CSF and serum (Qalb) is a sensitive parameter for the analysis of BCSFB integrity [11]: values less or equal to 0.01 indicate an intact BCSFB, whereas values > 0.01 are considered to occur only in defective BCSFB. In our study, Qalb was calculated at each observing point to assess the function of the BCSFB. Standardized turbidimetric assays (Cobas Integra^®^ Albumin, Roche^®^ Diagnostics, Mannheim, Germany) were used to determine the albumin levels in the CSF and the serum. The enrolled patients were divided into two groups to assess the influence of the BCSFB function on PMN elastase: group 1 with an intact and group 2 with a defective BCSFB.

### Data analysis

The Sigma Stat^®^ 3.0 software package (SPSS^®^ Inc., Chicago, USA) was used for all statistical analyses. Data were mostly provided in mean ± SEM. For non-parametric data, ANOVA (analysis of variance on ranks) according to Kruskal–Wallis was used to compare the values of the different groups. To analyze differences over the course of time, the Student–Neumann–Keuls test was applied after ANOVA. A *p* value < 0.05 was considered statistically significant.

## Results

### Patient population and clinical data

23 patients suffering from isolated severe TBI met the initial inclusion criteria. The mean age accounted for 46 ± 5 years. Eight patients died during the observation period of 72 h due to untreatable brain swelling and/or brain stem herniation and were, therefore, excluded. In total, 15 patients (5 females, 10 males) were finally enrolled in the study. Local or systemic infections were not observed during the observation period of 72 h. Among these 15 patients, three died later from their severe injuries (Table 1). The main reasons for TBI were either road traffic accidents or falling from a great height. Ten (5 females, 5 males) patients with a mean age of 40 ± 11 years were included in the control group. All patients had no records of CNS disease or trauma. In the control group, CSF was obtained while the patients received spinal anesthesia for a standard elective orthopedic procedure of the lower extremity.

**Table 1:** Demographic and clinical data of patients

BCSFB function	Patient no.	Age (years)	Sex	Injury	Intervention	Survival
Intact	I	32	f	SAH, SDH	EVD	Yes
Intact	II	82	m	SAH, SDH, longitudinal fracture of the petrous part of temporal bone, contusion bleeding	EVD	Yes
Intact	III	43	m	EDH, SAH	EVD, osteoclastic craniotomy, removal of hematoma	Yes
Intact	IV	23	m	SAH, contusion bleeding, impression fracture, intracranial foreign bodies	EVD, osteoclastic craniotomy	No
Intact	V	20	m	ICB, midface fracture	EVD, ORIF	Yes
Intact	VI	48	m	Cerebellar ICB, SDH	EVD, occipital craniotomy	Yes
Intact	VII	54	m	SAH	EVD	Yes
Intact	VIII	50	f	SAH, ICB, impression fracture	EVD, osteoclastic craniotomy	No
Intact	IX	34	m	SAH, longitudinal fracture of the petrous part of temporal bone, frontobasal fracture	EVD	Yes
Defective	X	50	m	ICB, SAH, skull base fracture, occipital bone fracture, orbital fracture	EVD	Yes
Defective	XI	27	f	SDH, SAH, contusion bleeding, impression fracture, temporal bone fracture	EVD, osteoclastic craniotomy	No
Defective	XII	41	f	SAH, SDH, skull base fracture	EVD	Yes
Defective	XIII	62	m	SDH, EDH, SAH, skull fracture	EV, osteoclastic craniotomy	Yes
Defective	XIV	67	m	EDH, ICB, SAH, skull base fracture, occipital bone fracture, fracture of the petrous part of temporal bone	EVD, osteoclastic craniotomy, removal of hematoma	Yes
Defective	XV	69	f	ICB, SAH	EVD, osteoclastic craniotomy	Yes

*EDH* epidural hematoma, *EVD* extraventricular drain, *ICB* intracerebral bleeding, *ORIF* open reduction and internal fixation, *SAH* subarachnoidal hemorrhage, *SDH* subdural hematoma

### Dynamics of the PMN elastase in the early posttraumatic phase

Over the entire observation period of 72 h a continuous increase of PMN elastase was found. Starting at levels of 4.97 ± 1.9 ng/ml on admission the increase resulted in 9.33 ± 1.7 ng/ml at 12 h after TBI, 27.12 ± 10.6 ng/ml at 48 h and 57.10 ± 21.5 ng/ml at 72 h after TBI.

**Fig. 1:**
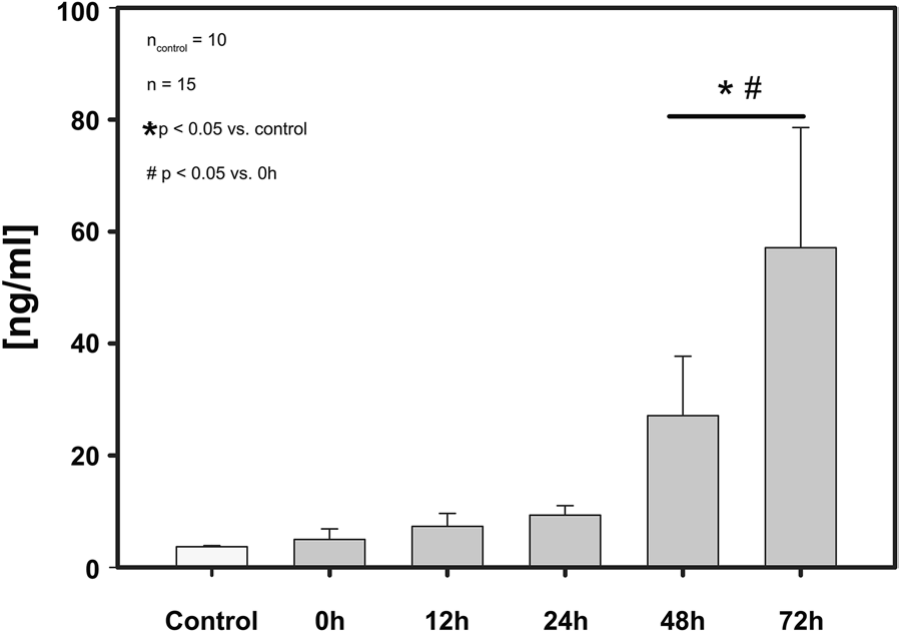
Dynamics of the PMN elastase in CSF. The *y*-axis shows the values of PMN elastase in ng/ml in the CSF (mean ± SEM). The respective sampling points are shown on the *x*-axis

The baseline level of the control group accounted for 3.68 ng/ml. Compared to the level measured immediately after admission (4.97 ng/ml), the first significant elevation of PMN elastase was registered 48 h after trauma (27.12 ng/ml at 48 h; *p* = 0.041). Similarly, the levels of PMN elastase at 48 (*p* = 0.042) and at 72 h (*p* = 0.036) after TBI (see above) were significantly higher compared to the baseline level of the control group (3.68 ng/ml). For more details, see Table 2 and Fig. 1.

**Table 2:** Dynamics of the PMN elastase in CSF

Time after trauma (h)	Control	T0	12 h	24 h	48 h	72 h
PMN elastase (ng/ml)	3.68 ± 0.3	4.97 ± 1.9	7.33 ± 2.3	9.33 ± 1.7	27.12 ± 10.6*^,#^	57.10 ± 21.5*^,#^

T0 = within 90 ± 45 min after admission; * *p* < 0.05 versus control group; ^#^ *p* < 0.05 versus study group at T0. PMN elastase in ng per ml in CSF (study group: *n* = 15; control group: *n* = 10; mean value ± standard error of the mean)

### Dynamics of PMN elastase on dependence of BCSFB function

At the time of admission, patients of group I (*n* = 9) with an intact BCSFBB showed a PMN elastase level of 2.93 ± 1.9 ng/ml and patients of group II (*n* = 6) with a defective BCSFBB presented a level of 8.80 ± 4.2 ng/ml. 12 h after TBI group I had a PMN elastase level of 3.87 ± 2.5 ng/ml while group II patients had a PMN elastase level of 11.72 ± 4.3 ng/ml. Group I patients showed a PMN elastase level of 8.2 ± 2.1 ng/ml at 24 h after TBI while group II patients presented a level of 9.80 ± 3.5 ng/ml. 48 h after trauma group I had a PNM elastase level of 14.56 ± 3.8 ng/ml and group II presented a level of 45.26 ± 26.6 ng/ml. At the observation end point of 72 h the elastase level of group I was 58.56 ± 32.6 ng/ml whereas the elastase level of group II patients accounted for 50.00 ± 27.1 ng/ml. In summary, both groups presented an increase of PMN elastase levels over time. Comparing the PMN elastase levels of groups I and II at the sample time points no significant difference could be found.

However, further analysis revealed significantly different PMN elastase levels in group I when comparing the level at time of admission to the levels at 48 as well as 72 h, respectively.

The control group presented a PMN elastase level of 3.68 ± 0.3 ng/ml. Comparing the control level to the PMN elastase values of group I and II significant differences were found at 48 h after TBI. The same findings resulted in comparing the control group level to patients’ levels with intact as well as defective BCSFBB at the 72-h time point. For a summary of values, see Table 3.

**Table 3:** Dynamics of the PMN elastase in CSF in dependence of the BCSFB function

Time after Trauma (h)	Control	T0	12 h	24 h	48 h	72 h
Group I PMN Elastase (ng/ml)	3.68 ± 0.3	2.93 ± 1.9	3.87 ± 2.5	8.2 ± 2.1	14.56 ± 3.8*^,#^	58.56 ± 32.6*^,#^
Group II PMN Elastase (ng/ml)	3.68 ± 0.3	8.80 ± 4.2	11.72 ± 4.3	9.80 ± 3.5	45.26 ± 26.6*	50.00 ± 27.1*

T0 = within 90 ± 45 min after admission; * *p* < 0.05 versus control group; ^#^ *p* < 0.05 versus same group at T0. PMN elastase in ng per ml in CSF (group I with intact BCSFB: *n* = 9; group II with defective BCSFB: *n* = 6; control group: *n* = 10; mean value ± standard error of the mean)

## Discussion

The actual study evaluated for the first time the dynamics of PMN elastase in cerebrospinal fluid of patients suffering from traumatic brain injury in the direct posttraumatic phase. PMN elastase levels were significantly elevated at 48 and 72 h compared to PMN levels at time of admission to the hospital as well as compared to baseline levels of the control group.

PMN elastase is involved in the shedding of tumor necrosis factor receptors from stimulated granulocytes [12]. In addition, the concentration of PMN elastase can be used as an activity marker for the function of granulocytes [4]. Therefore, the evaluation of the release kinetics of the PMN elastase is of high relevance regarding its involvement in secondary brain damage.

In our study, a comparison of PMN elastase levels in patients with intact and defective BCSFB showed no significant difference for the different observation time points. This might indicate that the influx of neutrophilic cells via the BCSFB was regulated in both groups. This would be in line with the literature—the BCSFB is seen as selective immune cell trafficking gate with active immune-regulating mechanisms [13, 14]. Several studies have explored the mechanisms of neutrophil migration over the BCSFB after TBI in animal models [13, 15, 16]. It has been shown that the BCSFB produces CXC chemokines [15] which have neutrophil chemotactic activity and modulate neutrophil trafficking [17]. Recently, it has been reported that the hormone arginine vasopressin (AVP) enhances the posttraumatic influx of neutrophils across the BCSFB via activating the c-Jun N-terminal kinase (JNK) [16].

Various mechanisms could serve as potential targets for therapeutic interventions to reduce brain tissue damage and to improve the functional neurological outcome of the patient. In this context, the in vivo inhibition of JNK in rats significantly reduced the production of neutrophil chemoattractants by the BCSFB and significantly reduced the influx of neutrophils across the BCSFB [16].

Very recently, the inhibition of PMN elastase with sivelestat (ONO 5046) gained attention in the treatment of ischemic spinal cord injury, traumatic spinal cord injury and ischemic brain injury [18–20]. The administration of sivelestat leads to a better outcome in animal models such as rabbits [19] and rats [18, 20]. Unfortunately, the literature does not provide any studies using sivelestat after TBI. However, a study on knockout mice reported that the PMN elastase has a central role in acute pathogenesis and chronic functional recovery after TBI of the immature brain [21]. This study also confirmed that the treatment of wild-type mice with the PMN elastase inhibitor ZN200,355 (AstraZeneca, London, UK) at 2-, 6- and 12-h post-TBI resulted in a reduction of cell death and oxidative stress [21]. The results of these studies point out that further research is necessary to explore the effects of PMN elastase inhibition in an adult animal model. For acute lung injuries, the positive effect of inhibiting PMN elastase had already been detected 30 years ago [22]. However, it had taken decades of basic research until sivelestat was approved in Japan for the treatment of acute lung injuries. Recent studies confirm benefits of sivelestat administration for patients after acute lung injury [23, 24]. Analogous to TBI research, this points out that further basic research concerning PMN elastase might be valuable and is necessary.

It is important to describe which other factors may have stimulated the release of PMN elastase in CSF. Systemic infections could have caused an increase in PMN elastase, but none were observed in our patients. Local infections, especially bacterial infections, could also have stimulated the release of PMN elastase. No evidence of local infection was found in our patient population. However, it cannot be completely ruled out that bacteria were present.

The presented study owns several limitations. First, the levels of PMN elastase were measured and significant elevations were found, but the reasons for the elevations were not further evaluated. However, this is the focus of our ongoing research. Since the placement of a ventricular drainage always leads to some kind of brain injury, an increase of the elastase levels might also be caused by the therapeutic intervention itself. However, by comparing the results of the patients to those of the control group, our results show that brain injury leads to a significant increase of PMN elastase. For future studies, a second control group, receiving an extraventricular drain for different reasons than for TBI, should be considered. Finally, groups I and II were both very small, hence the interpretation regarding BCSFB function should not be overestimated.

## Conclusions

This is the first study that demonstrates that the PMN elastase becomes significantly elevated 48 h and 72 h after TBI in patients. Assessment of the BCSFB could not reveal any significant influence of its function on the PMN levels. Future clinical studies should evaluate whether the elevation of PMN elastase affects not only the outcome of animals but also of patients. This is the focus of the current research of our study group.
